# Study protocol for a randomized, triple-blind, placebo-controlled trial of modified Ukgan-san in patients with Hwabyung and neurotic symptoms

**DOI:** 10.3389/fpsyt.2026.1826882

**Published:** 2026-07-21

**Authors:** Hui-Yeong Park, Seok-In Yoon, Yerim Jeon, Jiho Pyun, Kieun Lee, Jae-Hong Yoo, Sun-Yong Chung, Jong Woo Kim

**Affiliations:** 1 Department of Neuropsychiatry, College of Korean Medicine, Kyung Hee University, Seoul, Republic of Korea; 2 Department of Neuropsychiatry in Korean Medicine, Kyung Hee University Medicine Center, Seoul, Republic of Korea; 3 Department of Neuropsychiatry, Kyung Hee University Korean Medicine Hospital at Gangdong, Seoul, Republic of Korea

**Keywords:** herbal medicine, Hwabyung, modified Ukgan-san, neurotic symptoms, randomized controlled trial

## Abstract

**Background:**

Hwabyung is a culture-related anger syndrome characterized by chronically suppressed anger and somato-emotional symptoms. Modified Ukgan-san is widely prescribed for neurotic symptoms; moreover, it is recommended for anger-related manifestations in the clinical practice guidelines for Hwabyung. However, no randomized controlled trials evaluating its efficacy in patients with Hwabyung are currently available. This study aims to investigate the efficacy and safety of modified Ukgan-san in patients with Hwabyung presenting with neurotic symptoms.

**Methods:**

This will be a single-center, randomized, placebo-controlled, triple-blind, two-arm, parallel clinical trial. A total of 104 adult participants diagnosed with Hwabyung and presenting with neurotic symptoms will be recruited and randomly assigned in a 1:1 ratio to receive modified Ukgan-san extract granules or placebo. The intervention will be administered thrice daily for 4 weeks. The primary outcome is the change in Hwabyung symptom severity, measured using a validated self-report Hwabyung Scale. Secondary outcomes include anger, depression, anxiety, neurotic symptom-related indices, and heart rate variability. Assessments will be conducted at baseline and at weeks 2, 4, and 8. Data will be analyzed using linear mixed models according to the intention-to-treat principle.

**Discussion:**

We believe this trial will provide high-quality evidence on the effectiveness and safety of modified Ukgan-san in patients with Hwabyung and neurotic symptoms, addressing the gap between clinical recommendations and controlled trial evidence.

**Ethics and dissemination:**

This study was approved by the Institutional Review Board of Kyung-Hee University Oriental Medicine Hospital in Gangdong (KHNMCOH 2025-10-001-001). The results will be disseminated through publication in peer-reviewed journals.

**Trial registration:**

This study was registered with the Clinical Research Information Service (CRIS), Republic of Korea (No. KCT0011347; December 18, 2025).

## 1 Introduction

Hwabyung is a culture-related syndrome primarily reported in East Asian cultural contexts, particularly in Korea. The Korean Standard Classification of Diseases (KCD) formally classifies it under code U22.2 and is also described as a culture-bound syndrome in the ICD-10 of the World Health Organization.

Hwabyung is characterized by emotional symptoms such as feelings of unfairness and resentment, emotional suppression, and han (恨), a culturally specific form of accumulated sorrow or unresolved resentment. Patients also experience various somatic symptoms, including chest tightness, sensations of heat, the feeling of something rising in the chest, and a lump-like sensation in the throat or epigastrium. These symptoms typically occur during stressful life events (1).

When standardized diagnostic criteria are applied, the prevalence of Hwabyung in Koreans ranges between 4.2-13.3% (2–5). Treatment for Hwabyung is generally provided by Korean medicine doctors (1). Existing psychiatric classification systems have been refined primarily focusing on depression and anxiety; however, diagnostic frameworks that conceptualize anger as a core pathological emotion remain relatively underdeveloped. Anger has generally been described as a symptom that may occur in several psychiatric conditions, including personality disorders, trauma-related disorders, attention-deficit/hyperactivity disorder, and intermittent explosive disorder in the Diagnostic and Statistical Manual of Mental Disorders, Fifth Edition (6, 7). However, these descriptions have not led to the development of an independent diagnostic construct centered on anger.

Hwabyung may function as a model of anger-related psychopathology within a broader psychiatric framework beyond the Korean cultural context. Although Hwabyung retains culturally shaped expressions of distress, it also encompasses transdiagnostically understandable psychopathological features, including suppressed anger, emotional distress, somatic symptoms, and chronic stress responses. In this respect, Hwabyung shows phenomenological overlap with depressive disorders, anxiety disorders, somatic symptom-related disorders, and stress-related disorders. Furthermore, Hwabyung has been conceptualized as a model that explains the process from stressful life events to emotional responses as well as subsequent adaptation and maintenance factors (1). When cultural elements are appropriately adjusted, Hwabyung may serve as a theoretical framework for the conceptualization of anger-related disorders within a global psychiatric context (8).Neurotic symptoms may be defined as patterns of distressing, non-psychotic symptoms such as anxiety, phobic symptoms, and obsessive symptoms (9, 10). The concept of neurotic symptoms was widely used in earlier Western psychiatry; however, its use has gradually declined in major diagnostic systems since the publication of the Diagnostic and Statistical Manual of Mental Disorders, Third Edition, owing to the nonspecific nature of these symptoms and the ambiguity of their conceptual boundaries.

Although Hwabyung is characterized primarily by anger and feelings of resentment or injustice, it clinically presents with a broad range of accompanying features, including irritability, anxiety, tension, and a tendency toward somatization. Neurotic symptoms can be understood as a construct encompassing emotional instability and heightened reactivity to negative affect; the anger reactivity can then be considered as one of the manifestations within the construct. In this context, neurotic symptoms are frequently observed in patients with Hwabyung.

Ukgan-san (抑肝散, *Yokukansan* in Japanese) is a traditional herbal formula whose origin is generally traced to *Boyeongchwalyo* (保嬰撮要), a medical text written by the Ming dynasty physician Xue Kai and later published by his son Xue Ji. In this study, Ukgan-san was used to treat liver-related disorders in children. Subsequently, in Japan, during the early 20th century, the interpretation of this formula expanded and specific indications such as neurosis, neurasthenia, and hysteria were proposed (11). More recently, a systematic review conducted in Japan reported that Ukgan-san–related formulations demonstrate significant effects in reducing aggression and agitated emotional states (12).

The Korean Medicine Clinical Practice Guidelines for Hwabyung recommend the use of Ukgan-san for anger-related symptoms (1). However, the recommendation presented in this clinical practice guideline was based on expert consensus, and no studies investigating Ukgan-san in patients with Hwabyung, even in the form of case reports, have been reported to date. Therefore, rigorous empirical evidence supporting its use remains necessary.

Therefore, the present study aims to investigate whether modified Ukgan-san improves symptoms in patients with Hwabyung accompanied by neurotic symptoms compared to a placebo control.

## 2 Methods

### 2.1 Trial design

This study is a two-arm, randomized, triple-blind, placebo-controlled, parallel-group superiority trial. Participants will be randomly assigned in a 1:1 ratio to the experimental or the control group according to a randomization sequence generated using Microsoft Excel (Microsoft Corporation, Redmond, WA, USA). To ensure allocation concealment, the randomization sequence will not be disclosed to the enrollment personnel. After enrollment, the participants will be assigned to the experimental or control group according to a pre-generated randomization sequence.

The trial will comprise a 4-week intervention period followed by a 4-week follow-up period. Participants assigned to the experimental group will receive modified Ukgan-san for 4 weeks, whereas those assigned to the control group will receive a placebo identical in formulation but without the active ingredients for the same duration. Assessments will be conducted at four time points: baseline (T1), mid-treatment (T2), post-treatment (T3), and follow-up (T4).

**Figure 1:**
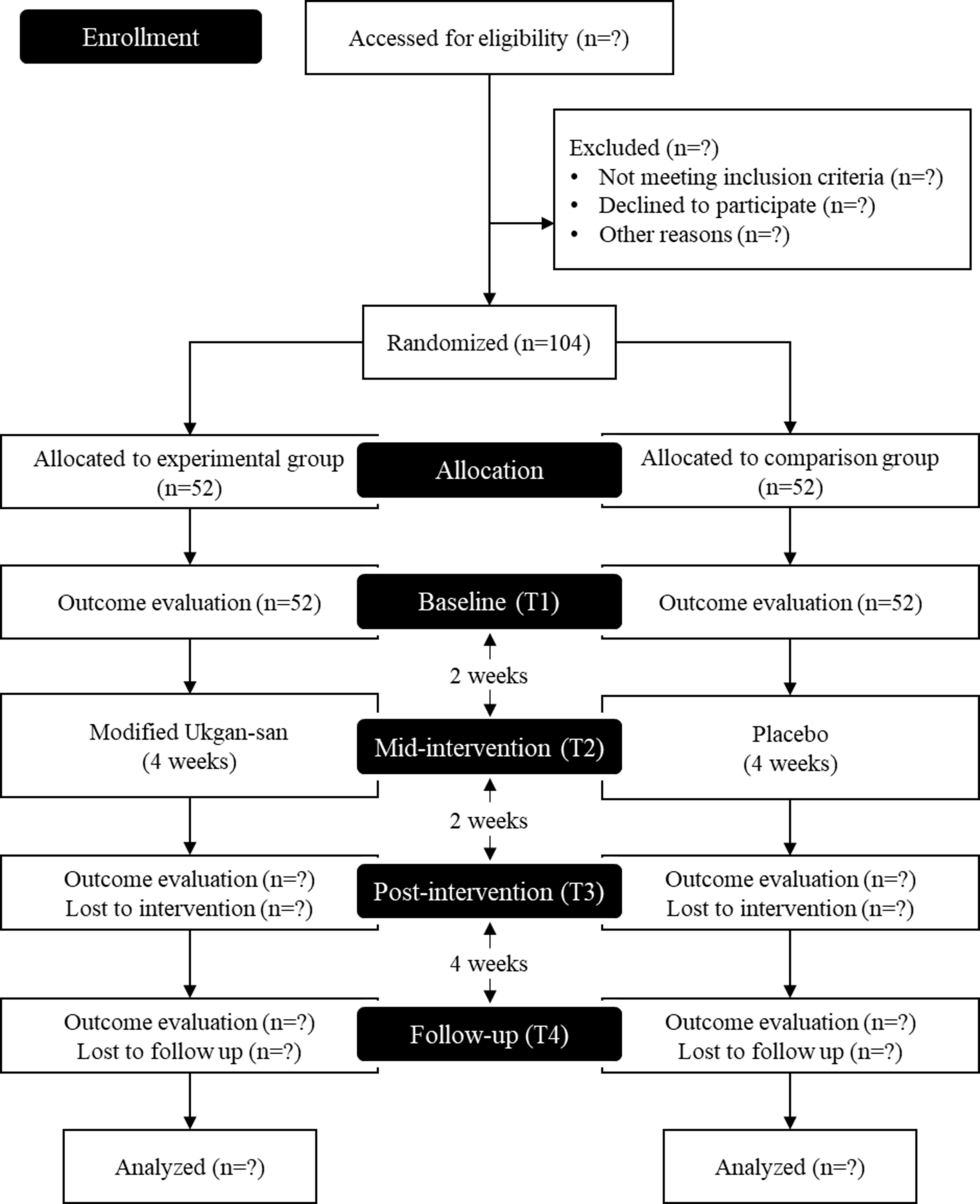
Study flow chart.

This trial was registered with the Clinical Research Information Service (CRIS) the Republic of Korea (KCT0011347). This trial complies with the SPIRIT guidelines and will be conducted in accordance with the tenets of the Declaration of Helsinki. **Figure 1** presents a flowchart that provides an overview of the study design.

The patients and the public were not involved in the design, conduct, or reporting of this trial, and no such involvement is planned.

### 2.2 Study setting

This trial will be conducted at Kyung Hee University Korean Medicine Hospital in Gangdong, South Korea. Participants will be recruited through advertisements in hospitals and subways. Potential participants interested in participating will be informed about the study. If they agree to participate, they will be screened according to the inclusion and exclusion criteria.

### 2.3 Eligibility criteria

Participants will be eligible for inclusion if they meet all the following criteria: 1) adults aged 19–65 years, of either sex; 2) a diagnosis of Hwabyung (U22.2) according to the Structured Clinical Interview for Hwabyung (SCID-HB), adapted from the SCID-IV format (1); 3) fulfillment of the diagnostic criteria for nervousness (R45.0), based on age- and sex-adjusted cutoff scores on the *Gyeong* (驚, alarm) and *No* (怒, anger) subscales of the Core Seven-Emotions Inventory Short Form (13); and 4) provision of written informed consent after receiving a full explanation of the study and agreeing to comply with all study-related procedures and precautions.

Participants will be eligible for exclusion if they meet all the following criteria: 1) assessed to be at risk of suicide; 2) currently diagnosed with or receiving treatment for schizophrenia, antisocial personality disorder, borderline personality disorder, histrionic personality disorder, or other severe personality disorders (including paranoid, schizoid, narcissistic, avoidant, or dependent personality disorders); 3) having a history of manic, hypomanic, or mixed episodes; 4) having current or past alcohol or other substance abuse or dependence; 5) having medical conditions that may influence the severity or manifestation of Hwabyung symptoms (e.g., hyperthyroidism); 6) having chronic diseases that are poorly controlled despite appropriate treatment (e.g., chronic active hepatitis, hypertension, or diabetes mellitus)^1^,^2^; 7) having hepatic or renal impairment, defined at screening as alanine aminotransferase (ALT), aspartate aminotransferase (AST), or alkaline phosphatase (ALP) levels ≥ 2 times the upper limit of normal, or blood urea nitrogen (BUN) or creatinine levels > 1.2 times the upper limit of normal; 8) having hereditary disorders such as galactose intolerance, Lapp lactase deficiency, or glucose–galactose malabsorption; 9) having diseases affecting drug absorption or gastrointestinal dysfunction following surgery that may impair drug absorption; 10) participation in another clinical trial within one month prior to study initiation; 11) a history of hypersensitivity or allergic reactions to the investigational product or any related medications; 12) current use of herbal medicines or crude herbal preparations other than the investigational product; 13) intellectual disability or cognitive impairment that could preclude understanding informed consent or compliance with study procedures; 14) pregnancy or breastfeeding; 15) being a woman of childbearing potential who did not agree to use approved contraceptive methods during the study period^3^; or 16) being deemed unsuitable for participation by the investigator for any other reason.

### 2.4 Intervention

Participants enrolled in the study will be randomly assigned to receive modified Ukgan-san or placebo for 4 weeks. The medication will be administered thrice daily after meals.

In addition, participants in both groups will be required to complete a daily anger diary. The anger diary records whether the participant experienced anger during the day, the number of anger episodes, the average intensity of anger (rated on a 0–10 scale, with higher scores indicating greater intensity), and the average duration of anger episodes (minutes). Participants will complete the diary each evening. Instructions on how to complete the anger diary will be provided by a blinded researcher.

#### 2.4.1 Description of the herbal formula and the placebo

The investigational drug used in this study was Gyeongjin Ukgansangajinpibanha Extract Granules (Korean: 경진억간산가진피반하엑스과립; Chinese characters: 抑肝散加半夏陳皮). This herbal preparation was approved by the Ministry of Food and Drug Safety (MFDS) of the Republic of Korea as an herbal medicine. According to the approved indications, it is effective for symptoms such as nervous irritability, insomnia, night crying in children, and *gamjeung* (疳症, a pediatric condition characterized by emaciation due to digestive dysfunction) in individuals with a weak constitution and heightened nervous excitability (14).

This formulation is based on Ukgan-san (抑肝散), which originates from Boying Chwaryo (保嬰撮要), a medical text written by the Ming dynasty physician Xue Kai (薛鎧). The original Ukgan-san prescription consists of *Angelica gigas* (當歸), *Uncaria sinensis* (釣鉤藤), *Cnidium officinale* (川芎), *Atractylodes japonica* (白朮), *Poria cocos* (茯苓), *Bupleurum falcatum* (柴胡), and *Glycyrrhiza uralensis* (甘草) (15). Ukgan-san and its related formulations have been increasingly investigated in clinical research and are widely used in Japan (11).

This formulation was selected for the present study for several reasons. First, the indications approved by the MFDS explicitly include neurotic symptoms, which correspond to the core symptom domain of Hwabyung. Second, a recent systematic review conducted in Japan reported that Ukgan san-related formulations significantly reduce aggression and excitatory emotional symptoms (12). Third, the Korean Medicine Clinical Practice Guidelines for Hwabyung recommend the use of Ukgan-san for patients whose primary symptom is anger (1). Gyeongjin Ukgansangajinpibanha Extract Granules were selected because they are the only commercially available modified Ukgan-san product currently marketed in Korea, allowing the use of a standardized and approved formulation. Therefore, this study hypothesized that this prescription would demonstrate therapeutic effects on both nervous irritability and the overall symptoms of Hwabyung.

The placebo was designed to be indistinguishable from the investigational drug in terms of formulation, appearance, packaging, and labeling.

#### 2.4.2 Composition of the herbal formula and the placebo

The study medication was manufactured by Gyeongjin Pharmaceutical Co., Ltd. in accordance with the formulation approved by the MFDS and the company’s standardized manufacturing protocol to ensure reproducibility and regulatory compliance. The research team did not intervene in the manufacturing process or modify product composition.

The placebo contained excipients identical to those used in the study medication, but excluded the active herbal ingredients, and was manufactured by Gyeongjin Pharmaceutical Co., Ltd. without any intervention from the research team.

The compositions of both products (product name, formulation, appearance, ingredients, and their amounts contained in the daily dose) are presented in **Table 1**.

**Table 1:** Details of the study medication.

Category	Study medication	Placebo
Product name	Ukgan-san-gajinpi-banha extract granules	Placebo
Dosage form and appearance	Light brown granules	Light brown granules
Ingredients and content (per 3 g single dose)	Active ingredients: Angelica gigas 1 g; *Uncaria rhynchophylla* 1 g; *Cnidium officinale* 1 g; *Atractylodes macrocephala* 1.33 g; *Poria cocos* 1.33 g; *Bupleurum falcatum* 0.67 g; *Glycyrrhiza uralensis* 0.5 g; *Citrus unshiu* peel 1 g; *Pinellia ternata* 1.67 g. Excipients: Lactose monohydrate 550 mg; Corn starch 1,290 mg	Formulated as a matched placebo for Ukgan-san-gajinpi-banha extract granules, containing excipients only (no active herbal ingredients). Excipients: Lactose monohydrate 750 mg; Corn starch 2,085 mg Coloring agent: Caramel 165 mg
Storage conditions	Stored in a tightly closed container at room temperature	Stored in a tightly closed container at room temperature
Retest period	36 months from the date of manufacture	Not applicable
Manufacturer	Gyeongjin Pharmaceutical Co., Ltd.	Gyeongjin Pharmaceutical Co., Ltd.

#### 2.4.3 Manufacturing process and quality control

Both the study medication and placebo were manufactured at Gyeongjin Pharmaceutical Co., Ltd. Facilities, which are certified for Good Manufacturing Practice (GMP).

The herbal materials were extracted by adding purified water at 8–10 times the weight of the crude herbs and heating at 100 °C for 3 hours. The extract was concentrated and dried to produce the granules.

Quality control was performed in accordance with the regulatory standards of the Republic of Korea, and the manufacturer approved the quality control procedures. Quality assessment included appearance inspection, identification tests, disintegration testing, particle size testing, content uniformity testing, assays of marker compounds, heavy metal testing, assay testing, microbial limit testing, airtightness testing, visual inspection, and verification of packaging and labeling. Batch-to-batch consistency was ensured through a standardized manufacturing process.

#### 2.4.4 Dosage and administration

Participants in both groups will take either the study medication or placebo three times daily after meals (morning, lunch, and dinner) with sufficient water for 4 weeks. Each dose consists of one sachet containing 3 g granules.

Each box contains 54 sachets (18 days’ supply), including a 4-day visit window. Participants will receive one box at the first visit and another box at the second visit, for a total of two boxes during the study period.

The study medication and placebo are manufactured and packaged by Gyeongjin Pharmaceutical Co., Ltd. in accordance with Good Manufacturing Practice (GMP) standards and supplied to the investigational site pharmacist. The instructions provided to participants, including directions for use, storage conditions, and precautions, are delivered by a blinded researcher.

The site pharmacist maintains a drug accountability record for all study medications used in the trial, documenting the quantity dispensed and returned, the date of dispensing to each participant, and the date of return.

At the end of the clinical trial, all unused study medications, including both undistributed and returned medications, will be collected and disposed by Gyeongjin Pharmaceutical Co., Ltd. The site pharmacist will organize the relevant pharmacy documentation and submit it to the investigator.

#### 2.4.5 Criteria for discontinuation or modification of study intervention

No dose modification of the allocated intervention will be performed as the study medication is an MFDS-approved marketed drug. However, the allocated intervention may be discontinued, and the participant may be withdrawn from the trial prematurely in the event of serious adverse events, or if continuation of the trial is considered inappropriate based on clinical judgment during the study.

#### 2.4.6 Strategies for monitoring treatment adherence

Participants will be asked to return any unused study medication at their next scheduled visit if any doses remain during the study participation period. Medication adherence will be assessed based on the quantity of medications returned.

#### 2.4.7 Concomitant treatments

Participants currently taking herbal medicines or botanical preparations other than the study medication will not be eligible to participate in this study. In addition, participants may be withdrawn from the clinical trial if they take medications or other substances that may affect the evaluation of study outcomes during the study or follow-up period without the instruction or approval of the investigator.

### 2.5 Sample size

This study is a confirmatory clinical trial designed to evaluate the efficacy of modified Ukgan-san compared with placebo in improving Hwabyung symptoms after 4 weeks of treatment. The primary outcome is the Hwabyung Comprehensive Test (HCT) score (16).

Because no previous pharmacological trials have used the HCT score, direct effect size estimates were unavailable. Therefore, the target effect size was determined based on prior randomized placebo-controlled trials of herbal medicines for emotional symptoms such as anxiety and depression, including studies using the Hamilton Anxiety Rating Scale (HAMA), Hamilton Depression Rating Scale (HAMD), and Depression Anxiety Stress Scales (DASS) (17–19). In these studies, standardized between-group effect sizes ranged from approximately d = 0.30 to 0.68, indicating moderate effects. Previous pharmacological studies in Hwabyung patients using the Hwabyung Symptom Scale (HB-S) reported minimal effect sizes (20, 21); however, these studies differed substantially in interventions, indications, and study design, and therefore were not considered appropriate references for the present trial.

Considering the expected therapeutic effects of modified Ukgan-san on neurotic symptoms and excitability, and conventional interpretations of clinically meaningful effects in anxiety and depression research, a target effect size of Cohen’s d = 0.60 was selected for this study.

Using a two-sided significance level of 0.05, statistical power of 80%, and an effect size of d = 0.60, the required sample size was calculated as 44 participants per group (total n = 88). Assuming a dropout rate of 15%, the final target sample size was set at 104 participants, with 52 participants allocated to each group.

### 2.6 Randomization and allocation concealment

Participants will be allocated to either the experimental or control group in a 1:1 ratio using a simple randomization method. The randomization sequence will be generated and maintained using Microsoft Excel by an independent researcher (HYP) who is not involved in participant recruitment, assessment, or data analysis. Access to the randomization sequence will be restricted to the designated study coordinator, and investigators responsible for participant enrollment and assessment will not have access to the allocation sequence.

The manufacturer will prepare the study medication according to the randomization schedule. The prepared study medication will be labeled with a serial number in the UKS-AA-ZZZ format, provided to the principal investigator, and managed by the designated study pharmacist. In this labeling system, AA indicates the site number and ZZZ indicates the sequential number assigned to participants, beginning from 001.

The correspondence table linking the randomization schedule with the serial numbers of the study medication will be sealed and stored in a manner that allows verification of whether the seal has been broken, and access will be restricted to designated study management staff only. When a medication request is received from the investigator for a registered participant, the study pharmacist will assign the study medication sequentially to the participant and will record and manage the participant identification code on the study medication serial number log.

### 2.7 Blinding procedures

This clinical trial is designed as a randomized, triple-blind, placebo-controlled, parallel-group study. To maintain triple blinding, the placebo will be identical to the study medication in terms of formulation, appearance, packaging, and labeling.

Blinding will be maintained for all personnel involved in the study, including the investigators, participants, clinical research coordinators, and data analysts, except for the independent researcher responsible for generating and managing the randomization schedule. To preserve participant blinding, information regarding whether the administered medication is the active treatment or a placebo will not be disclosed to participants until the completion of the clinical study period.

The principal and study investigators will be responsible for assessing participant eligibility, obtaining informed consent, assigning participant identification numbers, managing participant schedules, providing clinical care and consultation, and completing case report forms. Although they oversee the overall conduct of the study, they must remain unaware of the treatment allocation to each participant. Clinical research coordinators will perform routine clinical trial tasks, including participant scheduling and study coordination and will remain blinded to treatment allocation.

Blinding will be maintained throughout the study. However, unblinding may have been considered in cases of serious medical emergencies. In such situations, unblinding will be performed only if knowledge of the treatment assignment is necessary for the appropriate medical management of the participant. The procedures for unblinding are as follows:

If the principal investigator determines that unblinding is necessary, the treatment allocation will be revealed to confirm the participant’s assigned intervention. The procedures related to unblinding will be documented and managed by the researcher responsible for maintaining the randomization schedule.

If an emergency situation arises, in which it is impossible to follow the standard unblinding procedure and the investigator must break the blind immediately, unblinding may be performed without delay according to the predetermined procedures. In such cases, the investigator must document the circumstances and rationale for the unblinding.

### 2.8 Data collection and management

Assessments are scheduled at baseline (T1), mid-intervention (two weeks; T2), post-intervention (four weeks; T3), and follow-up (eight weeks; T4). Assessments include self-report ratings and heart rate variability (HRV) test. **Table 2** summarizes the assessment time points.

**Table 2:** Study assessment points.

Assessments	Screening	T1	T2	T3	T4
SCID-HB	○				
CEAQ-s	○				
Blood test (Liver and Kidney Function Tests)	○			○	
Blood test (Thyroid Function Tests)	○				
Urine Test (u-HCG)	○				
Demographic Information	○				
HCT		○	○	○	○
STAXI-K		○	○	○	○
K-BDI, BAI		○		○	○
K-DERS		○	○	○	○
HRV		○		○	○
Adverse Events			○	○	○
Concomitant Medications	○		○	○	○
Medication Adherence			○	○	

T1, baseline; T2, mid-intervention; T3, post-intervention; T4, follow-up; SCID-HB, Structured Clinical Interview for Hwabyung; CEAQ-s, the Core Seven-Emotions Inventory Short Form; HCT, Hwabyung Comprehensive Test; STAXI-K, State-Trait Anger Expression Inventory-Korean Version; K-BDI, State–Trait Anger Expression Inventory-Korean Version; BAI, Beck Anxiety Inventory; K-DERS, Difficulties in Emotion Regulation Scale–Korean version; HRV test, Heart rate variability test.

To minimize participant dropout, sufficient information regarding the study schedule, assessment time points, and participant responsibilities will be provided at the start of the study. Reminder text messages will be sent prior to each assessment to notify participants of upcoming visits and survey schedules. Upon completion of the study, the participants will receive compensation that has been approved in advance.

If the participants withdraw from the study, no further assessments will be conducted. Data will be analyzed according to the intention-to-treat principle using linear mixed models, which account for missing data without explicit imputation. The reasons for discontinuation (e.g., personal circumstances, poor adherence, or adverse events) will be documented and recorded.

The research staff responsible for administering the questionnaire assessments and heart rate variability (HRV) measurements will remain blinded and will provide participants with procedural instructions that do not influence their responses. To improve data quality, missing items and abnormal responses will be checked when questionnaires are collected. In addition, key outcome variables will be double-checked when transferring participant data into an electronic case report form (eCRF).

In this study, data will be managed using iCLICK, an electronic case report form (eCRF) system provided by the Korean Medicine Innovation Technology Development Program. The iCLICK system includes functions such as prevention of missing required fields, automatic detection of data entry errors, and management of time stamps and audit trails. These features support data quality assurance and enable systematic data security and monitoring.

Data obtained through the electronic case report form (eCRF) will be encrypted and stored on the researcher’s computer. Personally identifiable information will not be included in any of the publications. Personal information will be retained together with research data for a maximum of 10 years, after which it will be securely destroyed.

De-identified individual participant data, statistical codes, and other study materials will not be publicly available. Access may be provided only upon reasonable request from the corresponding author, and with institutional approval.

### 2.9 Monitoring

Because this study is an investigator-initiated clinical trial, only minimal monitoring will be conducted owing to practical resource constraints. The chair of the data monitoring committee will be the principal investigator who will oversee whether the monitoring process is conducted appropriately and will make the final decision regarding study termination. Another committee member will be a researcher responsible for monitoring the management and dispensing of the study medication. This researcher is aware of the purpose and design of the clinical trial but is not involved in the actual conduct of the study. Monitoring related to study medication management will be conducted through pharmacy visits every two months.

Owing to limited research resources, separate monitoring of the integrity of the source documents, case report forms (CRFs), and electronic case report forms (eCRFs) will not be performed. Instead, data consistency and entry errors will be minimized through field-level input validation (edit checks) and the automatic query generation functions for potential errors provided through the iCLICK system.

### 2.10 Primary outcomes

#### 2.10.1 HCT

The HCT (16) will be used to assess Hwabyung-related symptoms and psychological characteristics. This test comprises 13 items on Hwabyung symptoms, 5 items on the incident questionnaire, and 21 items on Hwabyung psychological characteristics. Hwabyung symptoms and psychological characteristics will be used to interpret scores. This test is a self-reported 5-point Likert scale. The total score ranges from 0 to 52 for Hwabyung symptoms, and from 0 to 84 for Hwabyung psychological characteristics. Higher scores indicate more severe Hwabyung symptoms and vulnerability. In a previous study (16), the Cronbach’s alpha for the HCT was 0.89 for Hwabyung symptoms and 0.95 for Hwabyung psychological characteristics.

In this study, the symptom subscale of the instrument will be designated as the primary outcome variable, and the psychological characteristics subscale will be treated as the secondary outcome variable.

### 2.11 Secondary outcomes

#### 2.11.1 State-trait anger expression inventory

The Korean Adaptation of the State-Trait Anger Expression Inventory (22) will be used to assess anger experiences and expression. This scale consists of 10 items on state anger, 10 items on trait anger, and 24 items on anger expression. This is a self-reported 4-point Likert scale. The total score ranges from 10 to 40 for state anger, 10 to 40 for trait anger, and 24 to 96 for anger expression. Anger expression comprises three factors: anger-out, anger-in, and anger control. Anger control is considered a functional expression of anger and will be reverse calculated. A higher score indicates experiencing and expressing more anger. In a previous study (22), Cronbach’s alpha was.90 for state anger, 0.75 for trait anger, 0.70 for anger-out, 0.66 for anger-in and, 0.79 for anger-control.

#### 2.11.2 Beck depression inventory

The Korean version of the Beck Depression Inventory (K-BDI) originally developed by Beck (23) and subsequently translated and validated for use in Korea by Rhee et al. (24) was used. The K-BDI is a self-report instrument designed to assess depressive symptoms and consists of 21 items rated on a 4-point scale (0–3). Higher total scores indicate greater severity of depressive symptoms. In a validation study (24), the internal consistency reliability was 0.85.

#### 2.11.3 Beck anxiety inventory

The Beck Anxiety Inventory (BAI), originally developed by Beck et al. (25), and subsequently adapted to Korean by Yook and Kim (26), will be used. The BAI is a self-report instrument designed to assess anxiety symptoms and consists of 21 items rated on a 4-point scale (0–3). Higher total scores indicate greater severity of anxiety symptoms. In a validation study (26), the internal consistency reliability was 0.91.

#### 2.11.4 Difficulties in emotion regulation scale

To assess neurotic symptoms, the Korean version of the Difficulties in Emotion Regulation Scale (K-DERS), originally developed by Gratz and Roemer (27) and subsequently translated and validated in Korean by Cho (28), will be used. Neurotic symptoms are closely associated with emotional sensitivity, hyperarousal, and impulsive responses, all of which reflect difficulties in emotional regulation. The DERS is a validated multidimensional instrument designed to assess these domains. Therefore, it is considered an appropriate measure to indirectly evaluate the characteristics of neurotic symptoms.

The scale consists of 35 items rated on a 5-point Likert scale (1–5). Higher total scores indicated greater difficulty in emotion regulation. In a validation study (28), the internal consistency reliability was reported as 0.92.

#### 2.11.5 Heart rate variability test

The HRV test is used to assess autonomic nervous system regulation. It consists of time-domain and frequency-domain analyses.

In the time-domain analysis, the following parameters will be measured: mean heart rate (MHR; average heart rate), standard deviation of NN intervals (SDNN; overall variability of heartbeats over the recording period), root mean square of successive differences (RMSSD; short-term variability in heart rate), and Physical Stress Index (PSI; an indicator of physical stress level).

In the frequency domain analysis, the following parameters will be assessed: total power (TP; overall autonomic nervous system activity), very low frequency (VLF; an additional sympathetic index), low frequency (LF; sympathetic activity index), high frequency (HF; parasympathetic activity index), LF norm, HF norm, and LF/HF ratio.

### 2.12 Adverse events

Adverse events will be assessed after each session of MQT-SH. The results section will describe the type, frequency, and severity of the adverse events. Severity will be assessed according to three grades (mild, moderate, or severe). Mild is defined as adverse reactions that does not interfere with activities of daily living. Moderate is defined as an adverse reaction that interferes with daily life but is not dangerous. Severe is defined as an adverse reaction that is severe and interferes with basic daily activities (e.g., eating and changing clothes).

In addition, blood tests will be performed to evaluate liver and renal function. After venous blood collection, the serum or plasma will be separated, and the following parameters will be quantitatively analyzed: liver function test parameters (aspartate aminotransferase [AST], alanine aminotransferase [ALT], total bilirubin, direct bilirubin, alkaline phosphatase [ALP], and gamma-glutamyl transferase [GGT]) and renal function test parameters (blood urea nitrogen [BUN] and creatinine).

All laboratory analyses will be conducted at the Department of Laboratory Medicine at the study site using standardized automated equipment and established protocols. The results will be reported with reference ranges and used to assess the presence of hepatic or renal dysfunction.

Approximately 10 mL of whole blood will be collected at the time of testing, and the specimens will be discarded immediately after confirmation of the results and completion of the screening procedures.

### 2.13 Statistical analyses

The primary outcome is the symptom subscale of the HCT, with higher scores indicating greater symptom severity. Treatment efficacy will be evaluated based on the changes from baseline to Week 4.

Between-group differences will be analyzed using a linear mixed model (LMM). Fixed effects will include group (intervention vs. control), time (baseline, Week 4, and Week 8), and the group × time interaction, with the subject included as a random intercept. The LMM was selected because it appropriately accounts for within-subject correlation in repeated-measures data and allows the inclusion of participants with incomplete data under the maximum likelihood estimation framework, thereby reducing potential bias compared to complete-case analysis.

All efficacy analyses will be conducted according to the intention-to-treat (ITT) principle, including all randomized participants. Missing data will be handled under the assumption of missing at random (MAR) using maximum likelihood estimation within the LMM framework. In this study, reasons for dropout and the occurrence of adverse events will be collected whenever possible, and the MAR assumption was considered appropriate because missingness may be associated with observable factors such as previous measurements, treatment response, and adverse events. In addition, a per-protocol (PP) analysis reflecting adherence and major protocol violations will be performed to evaluate the consistency of the results.

The primary analysis will test the significance of the group × time interaction. A pre-specified contrast will be used to estimate the between-group difference in the mean change at Week 4. A decrease in score represents clinical improvement; therefore, a negative between-group difference in change (Δ intervention − Δ control) that is statistically significant will be interpreted as evidence of treatment efficacy.

Secondary outcomes (anger, depression, anxiety, HRV, and neurotic symptoms) will be analyzed using the same LMM framework. Variables without intermediate measurements, such as depression, anxiety, and HRV, can also be analyzed with LMM; in these cases, random slopes will be omitted and only random intercepts will be included. Contrasts can be specified to examine between-group differences at each time point. To control for multiplicity across secondary endpoints, the p-values will be adjusted using the Holm procedure.

Safety outcomes will be assessed descriptively based primarily on adverse events at Weeks 2, 4, and 8, without formal statistical testing. Individual participants with values outside the normal range will be recorded and reported on a case-by-case basis.

All statistical tests will be two-sided, and the significance level will be set at p < 0.05.

## 3 Discussion

This study is a clinical trial designed to evaluate the efficacy and safety of modified Ukgan-san in patients with Hwabyung presenting with neurotic symptoms.

This study was designed to minimize the risk of bias by applying a triple-blind, placebo-controlled, randomized design. In particular, procedures were implemented to reduce expectancy effects and assessor bias that may occur in herbal medicine clinical trials, and the study protocol was developed with reference to the SPIRIT checklist and the RoB 2 assessment tool.

Hwabyung has been described as a culture-related psychiatric syndrome characterized by the coexistence of emotional and somatic symptoms. Accordingly, this study evaluates not only psychological outcomes, including depression, anxiety, anger, and neurotic symptoms, but also physiological outcomes such as heart rate variability (HRV) indices, in order to examine the effects of modified Ukgan-san on the symptoms of patients with Hwabyung from multiple perspectives.

In particular, this study focuses on anger, neurotic symptoms, and excitatory emotional states among the symptom domains of Hwabyung. The clinical practice guidelines for Hwabyung suggest, based on expert consensus, that Ukgan-san may be considered when anger-related symptoms are prominent (1). Therefore, this study aims to evaluate the applicability of modified Ukgan-san, which has been used to alleviate neurosis, neurasthenia, and agitated emotional states, in patients with Hwabyung.

In addition, this study is meaningful in that it explores the potential role of herbal medicine-based interventions as complementary and integrative approaches in the management of mental health conditions associated with Hwabyung. If the efficacy and safety of modified Ukgan-san are demonstrated, it may be considered a complementary therapeutic option for patients with Hwabyung presenting with anger and neurotic symptoms. Furthermore, by accumulating evidence-based interventions for culture-related psychiatric syndromes such as Hwabyung, this study may provide preliminary evidence for future research in the field of cross-cultural psychiatry.

This study has several limitations. First, there is a lack of clearly established diagnostic and evaluation criteria specifically for neurotic symptoms. Therefore, the subscales of the Core Seven-Emotions Inventory were collectively used as supportive reference measures, and improvements in neurotic symptoms were evaluated using the Difficulties in Emotion Regulation Scale. To address this limitation, specific symptom domains related to neurotic symptoms (e.g., depression, anxiety, and anger) were assessed concurrently. In addition, this study was conducted at a single center, and the intervention period was relatively short at 4 weeks. Furthermore, because Hwabyung is closely associated with Korean cultural contexts, caution is warranted when generalizing the findings to other cultural populations.

Future studies should include multicenter clinical trials to improve the generalizability of the findings and long-term follow-up studies to evaluate the durability and safety of modified Ukgan-san treatment. In addition, the development of standardized assessment tools for neurotic symptoms will be necessary.

### 3.1 Trial status

Recruitment and enrollment of participants has started.

## Footnotes

1 Poorly controlled hypertension is defined as blood pressure remaining above 140/90 mmHg despite treatment with an appropriate combination of three or more antihypertensive agents, including a diuretic, at adequate doses.

2 Poorly controlled diabetes mellitus is defined as persistent HbA1c ≥ 9.0% despite treatment with an appropriate combination of antidiabetic medications at adequate doses.

3 Double-barrier contraception refers to the use of a barrier method (e.g., condoms, diaphragm) in combination with another contraceptive method such as sterilization, intrauterine devices, or contraceptive creams, gels, or foams.

## References

[1] The Korean Society of Oriental Neuropsychiatry . Clinical practice guideline of korean medicine for hwabyung. Seoul: National Institute for Korean Medicine Development (2021). Available online at: https://nikom.or.kr/nckm/module/practiceGuide/view.do?guide_idx=147&menu_idx=14 (Accessed March 3, 2026).

[2] Min SK . Study of hwa-byung. Seoul: ML communications (2009).

[3] Min SK Kim JH . A study on Hwabyung in Bokil island. In: J korean neuropsychiatr assoc, vol. 25. (1986). p. 459–66. Available online at: https://www.riss.kr/link?id=A1995490 (Accessed March 3, 2026).

[4] Kim H-K Park J-Y . Prevalence and related factors of Hwabyung for the aged woman in rural community. In: J korean public health nurs, vol. 18. (2004). p. 234–42. Available online at: https://www.riss.kr/link?id=A100277725 (Accessed March 3, 2026).

[5] Lee J-G Lee J-H . Study on the prevalence of Hwa-Byung diagnosed by HBDIS in general population in Kang-won province. In: J orient neuropsychiatry, vol. 19. (2008). p. 133–9. Available online at: https://www.riss.kr/link?id=A82704172 (Accessed March 3, 2026).

[6] Lubke GH Ouwens KG de Moor MH Trull TJ Boomsma DI . Population heterogeneity of trait anger and differential associations of trait anger facets with borderline personality features, neuroticism, depression, attention deficit hyperactivity disorder (ADHD), and alcohol problems. Psychiatry Res. (2015) 230:553–60. doi: 10.1016/j.psychres.2015.10.003 26454404 PMC4655156

[7] American Psychiatric Association . Diagnostic and statistical manual of mental disorders. Washington, DC: American Psychiatric Publishing (2022).

[8] Min SK . Clinical correlates of hwa-byung and a proposal for a new anger disorder. Psychiatry Investig. (2008) 5:125–41. doi: 10.4306/pi.2008.5.3.125 20046356 PMC2796026

[9] Andrews G Stewart G Morris-Yates A Holt P Henderson S . Evidence for a general neurotic syndrome. Br J Psychiatry. (1990) 157:6–12. doi: 10.1192/bjp.157.1.6 2397364

[10] National Center for Biotechnology Information . Neurotic disorder. In: MedGen. Available online at: https://www.ncbi.nlm.nih.gov/medgen/10334 (Accessed March 3, 2026).

[11] Cho KH Kim TH Jin C Lee JE Kwon S . The literary trends of herbal prescription Ukgan-san and its application in modern traditional Korean medicine. J Korean Med. (2018) 39:17–27. doi: 10.13048/jkm.18021

[12] Matsuda Y Kishi T Shibayama H Iwata N . Yokukansan in the treatment of behavioral and psychological symptoms of dementia: a systematic review and meta-analysis of randomized controlled trials. Hum Psychopharmacol. (2013) 28:80–6. doi: 10.1002/hup.2286 23359469

[13] Kim J . A study on the validation and standardization of the CSEI-s (The Core Seven Emotions Inventory–short form) (dissertation). Iksan: Won Kwang University (2023). Available online at: https://www.riss.kr/link?id=T16832005. dissertation (Accessed March 3, 2026).

[14] Ministry of Food and Drug Safety . Gyeongjin Ukgan-san-gajinpi extract granules [drug information. Available online at: https://nedrug.mfds.go.kr/pbp/CCBBB01/getItemDetailCache?cacheSeq=201404211aupdateTs2026-02-03%2013:37:18.0b (Accessed March 3, 2026).

[15] Chinese Text Project . Boyeongchwalyo (保嬰撮要) (2016). Available online at: https://ctext.org/wiki.pl?if=gb&res=664599 (Accessed August 15, 2018).

[16] Choi SW Kim JW . Hwabyung comprehensive test (HCT). Seoul: Pakyoungsa (2023).

[17] Wang Y Jia Y Liu X Yang K Lin Y Shao Q . Effect of Chaihu-Shugan-San on functional dyspepsia and gut microbiota: a randomized, double-blind, placebo-controlled trial. J Ethnopharmacol. (2024) 322:117659. doi: 10.1016/j.jep.2023.117659 38151181

[18] Chen S Xu Z Li Y Wang T Yue Y Hou Z . Clinical efficacy of the Chinese herbal medicine Shumian capsule for insomnia: a randomized, double-blind, placebo-controlled trial. Neuropsychiatr Dis Treat. (2022) 18:669–79. doi: 10.2147/NDT.S349427 35378821 PMC8976492

[19] Birling Y Zhu X Avard N Tannous C Fahey PP Sarris J . Zao Ren An Shen capsule for insomnia: a double-blind, randomized, placebo-controlled trial. Sleep. (2022) 45:zsab266. doi: 10.1093/sleep/zsab266 34788454

[20] Choi WC . The effect of Sihogayonggolmoryeo-tang on anxiety of patients with hwa-byung: randomized, double-blinded, placebo-control trial (dissertation). Daejeon: Daejeon University (2015). Available online at: https://www-riss-kr-ssl.openlink.khu.ac.kr/link?id=T13735319. dissertation (Accessed March 3, 2026).

[21] Kim SH . The effect of Bunsimgi-eum (Fenxinqiyin) on the chest discomfort of hwa-byung’s major symptom: randomized, double-blinded, placebo-control trial (dissertation). Daejeon: Daejeon University (2010). Available online at: https://www-riss-kr-ssl.openlink.khu.ac.kr/link?id=T11960347. dissertation (Accessed March 3, 2026).

[22] Chon KK Kim DY Yi JS . Development of the STAXI-K: IV. In: Korean J art ther, vol. 7. (2000). p. 33–50. Available online at: https://www.riss.kr/link?id=A105481542 (Accessed March 3, 2026).

[23] Beck AT . Depression: clinical, experimental and theoretical aspects. In: Harper & Row, New York (1967).

[24] Rhee MK Lee YH Park SH Sohn CH Chung YC Hong SK . A standardization study of Beck Depression Inventory I–Korean version (K-BDI): reliability and factor analysis. In: Korean J psychopathol, vol. 4. (1995). p. 77–95. Available online at: https://kiss-kstudy-com-ssl.openlink.khu.ac.kr/Detail/Ar?key=1809835 (Accessed March 3, 2026).

[25] Beck AT Epstein N Brown G Steer RA . An inventory for measuring clinical anxiety: psychometric properties. J Consult Clin Psychol. (1988) 56:893–7. doi: 10.1037/0022-006X.56.6.893 3204199

[26] Yook SP Kim ZS . A clinical study on the Korean version of Beck Anxiety Inventory: comparative study of patient and non-patient. In: Korean J clin psychol, vol. 16. (1997). p. 185–97. Available online at: https://scienceon.kisti.re.kr/srch/selectPORSrchArticle.do?cn=ATN0050379485&SITE=CLICK (Accessed March 3, 2026).

[27] Gratz KL Roemer L . Multidimensional assessment of emotion regulation and dysregulation: development, factor structure, and initial validation of the difficulties in emotion regulation scale. J Psychopathol Behav Assess. (2004) 26:41–54. doi: 10.1023/B:JOBA.0000007455.08539.94

[28] Cho YR . Assessing emotion dysregulation: psychometric properties of the Korean version of the difficulties in emotion regulation scale. In: Korean J clin psychol, vol. 26. (2007). p. 1015–38. Available online at: https://www.dbpia.co.kr/Journal/articleDetail?nodeId=NODE11092815 (Accessed March 3, 2026).

